# Performance Gains in Genome-Wide Association Studies for Longitudinal Traits via Modeling Time-varied effects

**DOI:** 10.1038/s41598-017-00638-2

**Published:** 2017-04-04

**Authors:** Chao Ning, Huimin Kang, Lei Zhou, Dan Wang, Haifei Wang, Aiguo Wang, Jinluan Fu, Shengli Zhang, Jianfeng Liu

**Affiliations:** National Engineering Laboratory for Animal Breeding; Key Laboratory of Animal Genetics, Breeding and Reproduction, Ministry of Agriculture; College of Animal Science and Technology, China Agricultural University, Beijing, 100193 China

## Abstract

Complex traits with multiple phenotypic values changing over time are called longitudinal traits. In traditional genome-wide association studies (GWAS) for longitudinal traits, a combined/averaged estimated breeding value (EBV) or deregressed proof (DRP) instead of multiple phenotypic measurements per se for each individual was frequently treated as response variable in statistical model. This can result in power losses or even inflate false positive rates (FPRs) in the detection due to failure of exploring time-dependent relationship among measurements. Aiming at overcoming such limitation, we developed two random regression-based models for functional GWAS on longitudinal traits, which could directly use original time-dependent records as response variable and fit the time-varied Quantitative Trait Nucleotide (QTN) effect. Simulation studies showed that our methods could control the FPRs and increase statistical powers in detecting QTN in comparison with traditional methods where EBVs, DRPs or estimated residuals were considered as response variables. Besides, our proposed models also achieved reliable powers in gene detection when implementing into two real datasets, a Chinese Holstein Cattle data and the Genetic Analysis Workshop 18 data. Our study herein offers an optimal way to enhance the power of gene detection and further understand genetic control of developmental processes for complex longitudinal traits.

## Introduction

Genome-wide association studies (GWAS) have become a powerful tool to pinpoint genetic variation of complex traits in livestock, plants, humans and model organisms. Linear mixed models (LMM) have been widely applied in GWAS as they performed well in correcting environmental factors, controlling population stratification and accounting for relatedness between individuals^1–6^.

So far, most of these commonly-used methods have been focusing on typical phenotypic data where single record per individual is collected. However, a different type of phenotypic data generated from longitudinal traits has seldom received attentions in GWAS. Longitudinal traits belong to a type of complex traits measured at various time points during a life cycle, such as blood pressures, daily gain, milk production, and residual feed intake, *etc*. Analyzing such kind of data affords us an opportunity to investigate the heterogeneity of traits over time and early prediction of longitudinal traits or diseases^7, 8^.

In previous quantitative trait loci (QTL) linkage analysis on longitudinal traits, three statistical strategies are proposed as follows: The first one is based on repeatability model or multivariate model, which treats the multi-point measured trait as repeated measurements of the same trait or as different traits^9, 10^. The second one is based on phenotypic combination where multi-point measures of each individual are firstly fitted by some smoothing methods, and the estimated curve parameters, accumulated or average values for a period of time are then used as the alternative response variables^11–13^. The third one is based on varying coefficient model, which fits the coefficients of genetic and environmental effects as the linear regression on a set of splines or polynomials of time to model the time-varied effects^14–17^.

These methods aforementioned have respective limitations. Specifically, repeatability/multivariate model is unable to explore time-dependent relationship between successive measures, and multivariate model is often difficult to apply in practice because it fits too many parameters in the model^18^. The strategy of phenotypic combination merely works when all effects are supposed to be constant over time^19^. Varying coefficient model is only suitable to well-structured dada where all individuals must be measured at the fixed time points. The drawbacks for these strategies limit their further application in the GWAS.

In dairy cattle breeding, estimated breeding values (EBVs) or deregressed proofs of EBV (DRPs) are preferred as the response variable of GWAS^20, 21^. Nevertheless, it has been indicated that EBVs incorporating familial information would lead to higher false positive rates (FPRs)^22^. DRPs had adjusted for parental average effect^23^, but could still lead to higher FPRs when the EBVs were the results of repeated measurements^22^. In human disease studies, the interaction between Single Nucleotide Polymorphism (SNP) and time/age was incorporated in the analysis model^24–26^. However, Wu and Lin indicated that there were various dynamic patterns of genetic control (permanent QTLs, early QTLs, late QTLs and inverse QTLs)^27^, which could not be completely explained by the interaction model.

Random regression model^28^ provides a better way to model the time-varied measurements/traits, and has been widely used in genetic evaluation of dairy cattle^29, 30^. Recent studies have proved that it increased the power to detect QTL compared with the combining phenotypes strategy, repeatability model and multivariate model in QTL mapping^18, 19^. Random regression model was also suitable for QTL detecting in the presence of gene by environment interactions^31^. However, application of this model in GWAS has not been fully surveyed so far.

In this study, we developed two models based on random regression model to model the time-varied SNP effect for the GWAS analysis, *i.e*., functional GWAS model treating each SNP as the covariate (fGWAS-C) and functional GWAS model treating each SNP as the factor (fGWAS-F). A series of simulation studies were performed to investigate the properties of the proposed models, and to compare with previous developed models, *i.e*., genome-wide association studies where EBVs or DRPs were used as response variable with polygenic effects modelled (GWAS-EBV-P or GWAS-DRP-P), genome-wide association studies where EBVs or DRPs were used as response variable without polygenic effects modelled (GWAS-EBV-NP or GWAS-DRP-NP), and genome-wide association studies where estimated residuals were used as response variable (GWAS-Residual). We further validated our model with a Chinese Holstein cattle data and the Genetic Analysis Workshop 18 (GAW18)^32^.

## Results

### Simulations

#### Comparison of false positive rates

**Figure 1:**
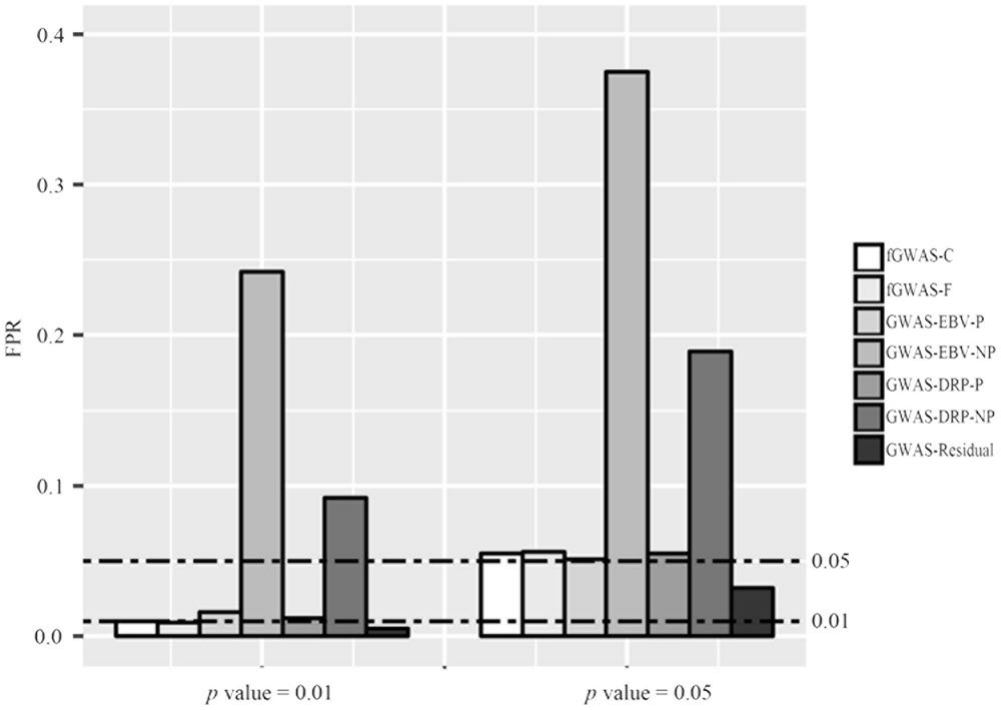
The type I errors (false positive rates, FPRs) of different GWAS models for the simulated data at the tabulated thresholds of *p* = 0.05 and *p* = 0.01 for the simulation study.

The FPRs (obtained by comparison with tabulated thresholds of *p* value = 0.01 and 0.05) of the evaluated models were shown in Fig. 1. As the FPRs were independent of the QTN heritability (the proportion of phenotypic variance explained by a single QTN) in the simulation (see Materials and Methods), we averaged the FPRs across different QTN heritabilities (${h}_{{\rm{QTN}}}^{2}$ = 0.1%, 0.5%, 1% and 2%).

Our results indicated that FPRs of proposed fGWAS-C and fGWAS-F models as well as traditional GWAS-EBV-P and GWAS-DRP-P models were very close to the tabulated thresholds of 0.01 and 0.05, denoting these models could be used to detect QTN underlying longitudinal traits with reasonable FPRs.

It is notable that both GWAS-EBV-NP and GWAS-DRP-NP models resulted in a clearly higher FPRs compared with other models, which was in agreement with the findings of Ekine *et al*.^22^. This was due to failures of reflecting genetic relationship among experimental individuals in the models. GWAS-Residual model rendered relatively conservative FPRs among all models investigated, which further verified the similar findings of GRAMMAR^5, 22, 33^.

#### Power comparison

**Figure 2:**
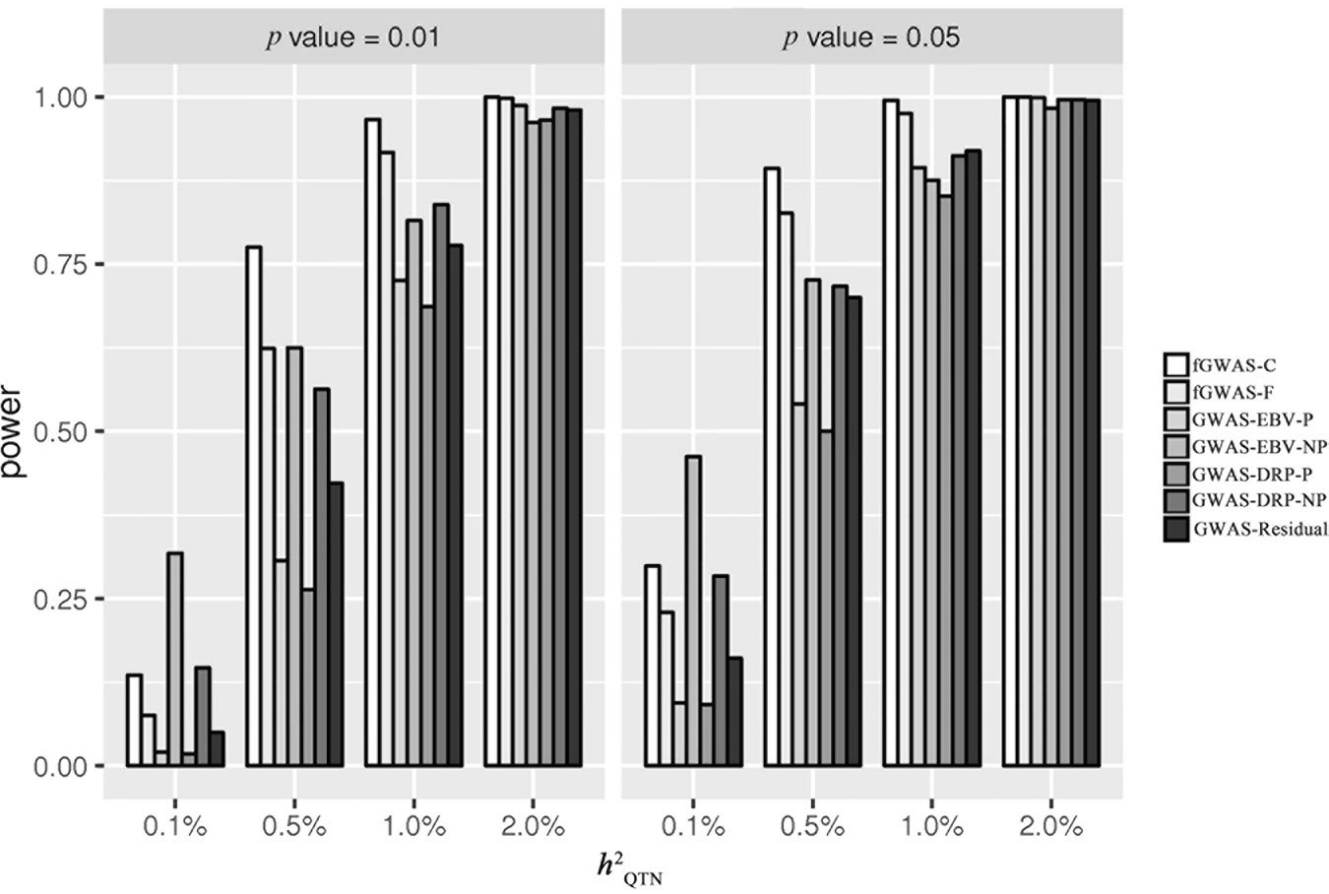
The powers of different GWAS models with alternative QTN heritabilities at tabulated thresholds of *p* = 0.01 and *p* = 0.05 for the simulation study.

Figure 2 showed the powers of QTN detection corresponding to seven different models under each scenario with different QTN heritabilities. In general, the powers of all methods improved with the increase of QTN heritability. Especially when the QTN heritability reached 2%, the powers were very close to 100% for all methods.

As expected, our proposed models (fGWAS-C and fGWAS-F) achieved the highest power among all the models employed under all scenarios except when ${h}_{{\rm{QTN}}}^{2}=0.1 \%$, where the GWAS-EBV-NP and GWAS-DRP-NP models achieved a higher power at the cost of high FPRs. It should be pointed out that the GWAS-Residual model also obtained relatively higher power even under the relatively conservative circumstance. For our two models, the model fGWAS-C achieved more power than fGWAS-F. The advantage of fGWAS-F was that it could test additive and dominant effect simultaneously. But it could lose power for testing the additive effect in some degree. Interestingly, we found that the GWAS-EBV-P gained more power than GWAS-DRP-P in our simulation.

We also evaluated powers of all models using empirical thresholds based on different FPRs for null-effect SNP. The receiver operating characteristic (ROC) curves plotting the statistical powers against FPRs were shown in Figures S1 and S2. The curves indicated that the fGWAS-C model performed best at all levels of QTN heritabilities, and fGWAS-F model was the second best except it achieved a slightly lower power than GWAS-Residual model at a lower QTN heritability (0.1%).

**Figure 3:**
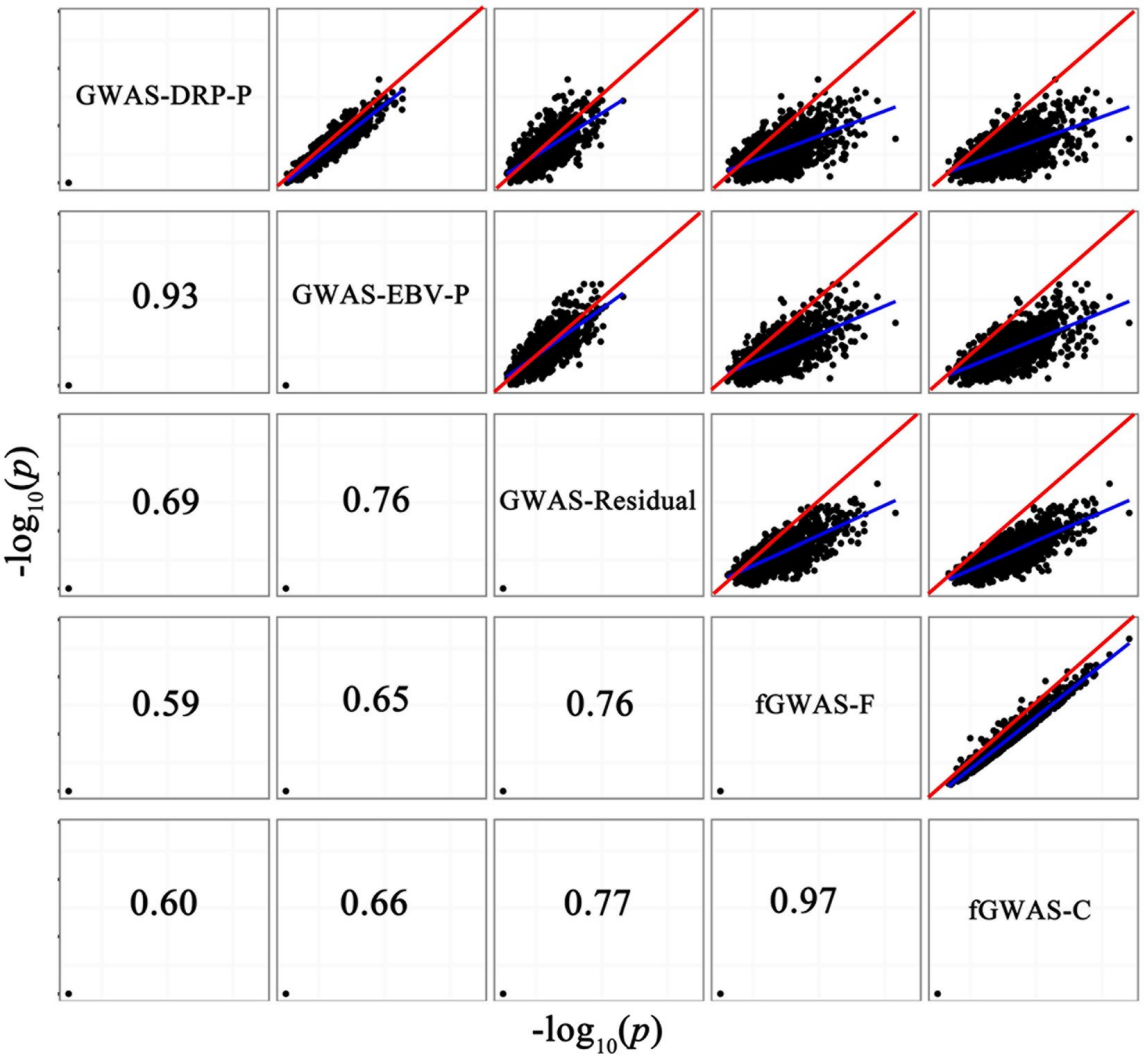
Comparison of *p*-values ($-{\mathrm{log}}_{{\rm{10}}}({p})$) using different GWAS models at QTN heritability of 1.0% for the simulation study. Scatterplots of $-{\mathrm{log}}_{10}(p)$ for any two GWAS models were shown at the upper triangular, with Pearson correlation coefficients listed at the lower triangular. The read lines represented regression line y = x; the blue lines were the lines of best fit for $-{\mathrm{log}}_{10}(p)$ of each two models.

Furthermore, we discovered that the *p*-values ($-{\mathrm{log}}_{10}(p)$) between our two proposed models as well as the *p*-values for the GWAS-EBV-P and GWAS-DRP-P models were strongly correlated (r > 0.9) (Fig. 3). This indicated that these two pairs of models could lead to similar orders of *p* values, respectively.

#### Estimation accuracy of functional QTN effect

**Figure 4:**
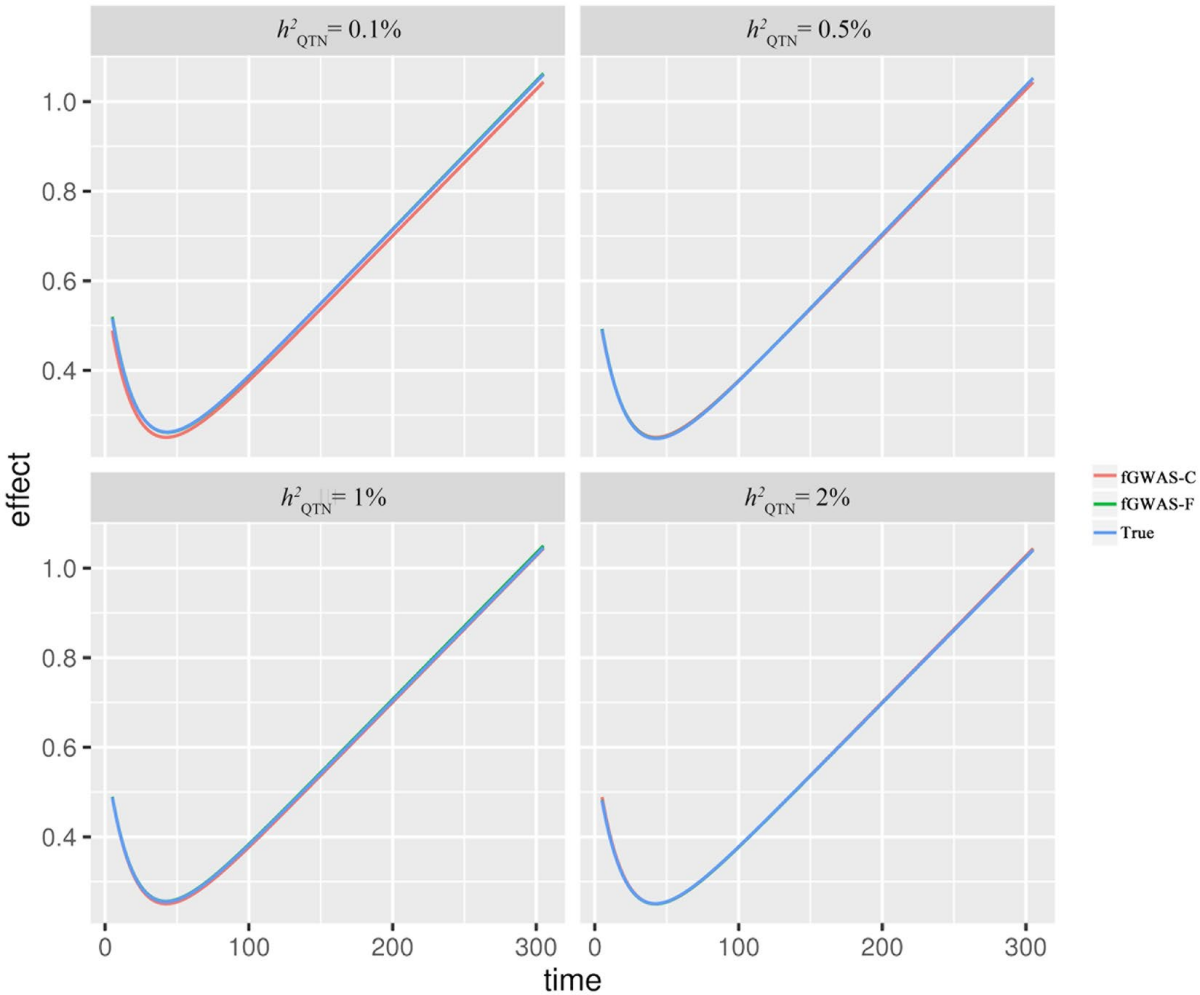
The plots of average additive genetic effect curves predicted by the fGWAS-C and fGWAS-F models against the simulated true curves with alternative QTN heritabilities for the simulation study.

The average of estimated cumulative additive effect (see equation S3 in Supplementary Methods for calculation) or estimated cumulative effect of the QTN for different models and their corresponding standard deviations (SD) and root-mean-square errors (RMSE) were summarized in Table 1. As no dominant effect was simulated for the causal QTN, the cumulative dominant effect predicted by fGWAS-F was very close to zero (Table S1). In the simulation, the true cumulative QTN effect was fixed at 175.21. It could be seen that the fGWAS-C and fGWAS-F models achieved the most accurate estimate of the QTN effect regardless of QTN heritability, while the other models always underestimated the true QTN effect in different degree. Meanwhile, the standard deviations of the cumulative effect estimated by all the models decreased as the QTN heritability increased except the GWAS-Residual model, which implied that a more accurate estimation of QTN effect could be realized at a higher QTN heritability. The root-mean-square-errors of our two proposed models were always the smallest across all models for each QTN heritability scenario, and they were very close to the corresponding standard deviations for these two models. Furthermore, the average additive genetic effect curves across the 1,000 replicates predicted by the fGWAS-C and fGWAS-F models shared perfect concordance with the true curves (Fig. 4).

**Table 1:** Means, standard deviations (SD), and root-mean-square errors (RMSE) of estimated cumulative additive genetic effect of the QTN for different GWAS models with various QTN heritabilities in the simulation study.

Models	*h* ^2^_QTN_							
	0.1%		0.5%		1%		2%	
	Mean ± SD	RMSE	Mean ± SD	RMSE	Mean ± SD	RMSE	Mean ± SD	RMSE
fGWAS-C	179.45 ± 135.33	135.33	175.9 ± 63.54	63.52	176.97 ± 48.09	48.10	174.76 ± 34.17	34.15
fGWAS-F	182.87 ± 142.99	143.12	177.5 ± 63.55	63.56	175.04 ± 47.53	47.50	176.1 ± 34.26	34.26
GWAS-EBV-P	29.17 ± 41.67	151.87	34.97 ± 16.59	141.22	35.84 ± 11.62	139.86	35.19 ± 7.28	140.21
GWAS-EBV-NP	71.52 ± 114.81	154.66	67.92 ± 52.39	119.39	69.81 ± 37.35	111.82	69.73 ± 24.71	108.34
GWAS-DRP-P	71.65 ± 109.83	150.91	97.95 ± 48.75	91.34	108.85 ± 37.93	76.43	119.37 ± 27.21	62.11
GWAS-DRP-NP	108.00 ± 147.73	162.24	121.61 ± 69.30	87.58	132.73 ± 52.31	67.37	143.59 ± 36.18	48.04
GWAS-Residual	1.46 ± 1.08	173.75	6.40 ± 2.30	168.82	11.74 ± 3.25	163.50	21.53 ± 4.33	153.74

### Chinese Holstein cattle data

**Figure 5:**
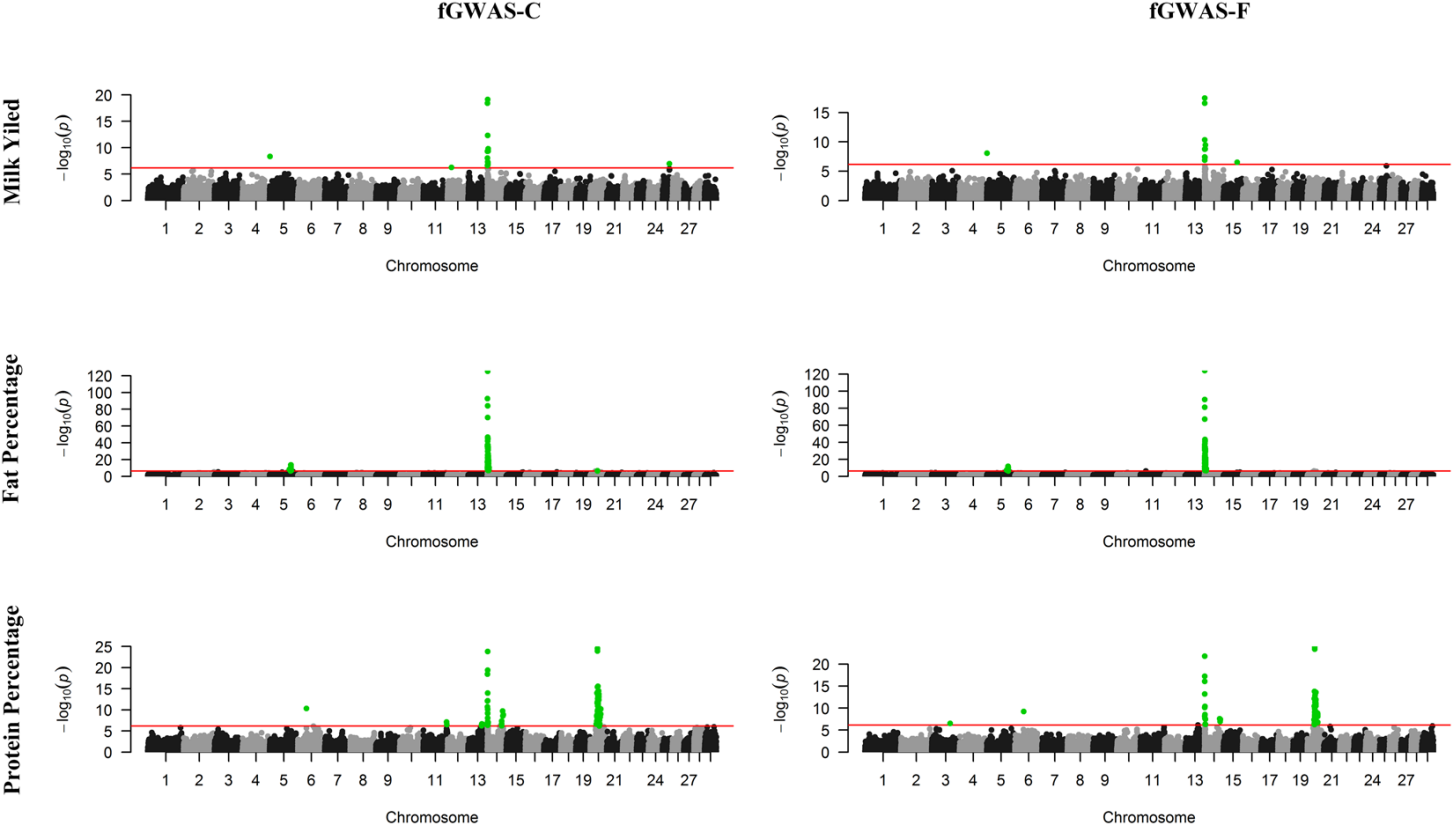
Manhattan plots of *p*-values for milk yield (MY), fat percentage (FP), and protein percentage (PP) by the fGWAS-C and fGWAS-F model for the Chinese Holstein cattle data. Chromosomes 1–29 were shown with black and grey intervals. The red horizontal lines indicated the genome-wise significance level of −log_10_(6.98 × 10^−7^) and SNPs above the lines were highlighted in green.

We used Akaike information criterion^34^ (AIC) as well as Bayesian Information Criterion^35^ (BIC) to determine the orders of basis functions. After model selection with AIC and BIC values, the model with a fifth-order basis functions for population mean, a third-order for additive genetic effects and a fifth-order for permanent environmental effects was best fit to the data for all the three traits (Table S2). Manhattan plots of $-{\mathrm{log}}_{10}(p)$ for milk yield (MY), fat percentage (FP) and protein percentage (PP) by the fGWAS-C and fGWAS-F models were shown in Fig. 5. For the three traits of Chinese Holstein cattle population, we found 215 genome-wise significant SNPs in total by our fGWAS-C and fGWAS-F models (Figure S3A). Among the 215 SNPs, 179 were commonly detected by both methods, while 33 and three were solely detected by fGWAS-C and fGWAS-F, respectively (Figure S3B). The results indicated that fGWAS-C and fGWAS-F shared perfect concordance, and fGWAS-F could lose power in some degree. Furthermore, 11 of these 215 SNPs, located in a relatively narrow segment (from 1.65 to 4.36 MB) of chromosome 14, were discovered to affect all the three traits. The well-known *DGAT1* (diacylglycerol O-acyltransferase 1) gene, reported to be a major gene affecting milk production traits^36^, is located within this region.

For milk yield, 3 of 17 significant SNPs were located on chromosome 5, 12 and 15, respectively, while all the remaining SNPs were located between 1.48 and 4.36 MB of chromosome 14. For fat percentage, 126 significant SNPs were found, and most of which were located between 91.13 and 95.74 MB on chromosome 5 (15 SNPs) and between 1.41 and 8.45 MB on chromosome 14 (106 SNPs). For protein percentage, most of the total 113 significant SNPs were located in the region 1.65–4.47 MB on chromosome 14 (24 SNPs), 28.80–38.49 MB on chromosome 20 (62 SNPs), and 44.16–45.87 MB on chromosome 20 (7 SNPs). Meanwhile, the majority of genome-wise significant SNPs (14 of 17 for milk yield, 103 of 126 for fat percentage and 88 of 113 for protein percentage) were located within reported QTLs for three corresponding traits. Interestingly, two regions (93.13–95.74 MB on chromosome 5 for FP, 44.16–45.87 MB on chromosome 20 for PP) were not overlapped with the known QTL regions and could be potential candidate QTL regions influencing the milk traits. QTL information of the three traits was obtained from Animal QTL Database (QTLdb; http://www.animalgenome.org/QTLdb). The detailed information of SNPs showing significant associations with the three traits, including their positions in the genome, *p* values, detected model, the nearest known genes and the PudMed IDs for nearest QTLs, were given in Tables S3 through S5.

The top significant SNP for the three traits was SNP ARS-BFGL-NGS-4939, which was located within *DGAT1* gene region. This SNP explained 1.45%, 13.72% and 1.93% of the phenotypic variation for milk yield, fat percentage and protein percentage with the fGWAS-F model, respectively. The curves of additive effects, dominance effects and QTL heritabilities of this SNP for three traits were shown in Figure S4.

### GAW18 data

**Figure 6:**
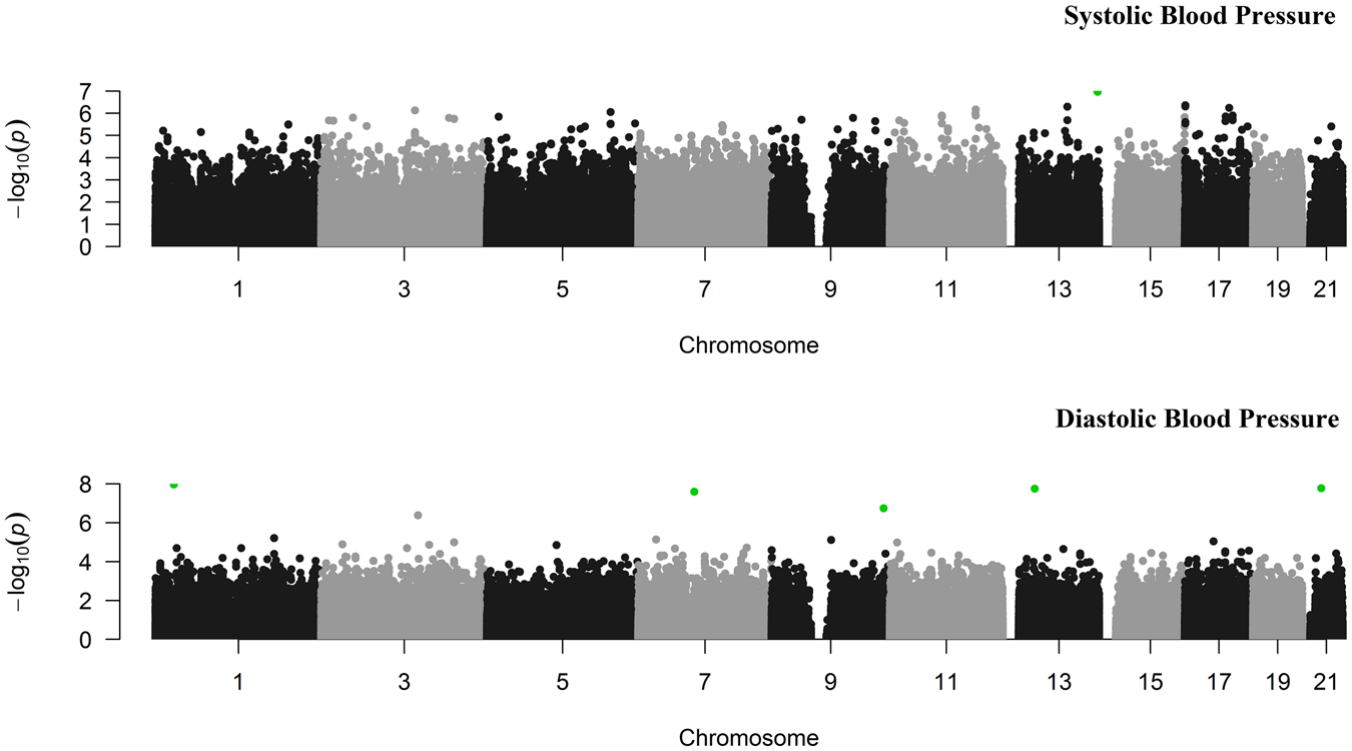
Manhattan plots of *p* values for systolic blood pressure (SBP) and diastolic blood pressure (DBP) by the fGWAS-F model for the GAW18 data. Odd numbered autosomes were shown with black and grey intervals. The significant SNPs (*q* values < 0.05) were highlighted in green.

As higher order basis functions did not converge, the model with a second-order basis functions for all the time-varied effects was used to fit GAW18 data. Manhattan plots of *p* values for two traits by the fGWAS-F model were shown in Fig. 6. For systolic blood pressure, two SNPs (on Chr13) reached the genome-wide significance level. Both of them are located within the region of gene *CUL4A*, which participates in the biological processes including nucleotide-excision repair, DNA damage recognition and regulation of DNA damage checkpoint. For diastolic blood pressure, 6 SNPs showed the genome-wide significance. The nearest genes to these 6 SNPs are *CDC42* (within), *TMEM248* (within), *RN7SL43P* (782 bp away), *VAV2* (within), *UFM1* (53 kb away), and *AP000959.2* (1.47 Mb away), respectively. Interestingly, both *CDC42* and *VAV2* genes participate in the biological process of blood coagulation, and *CDC42* gene also participates in heart contraction.

## Discussion

Recently, a growing number of studies indicated that the expression of genes was time-dependent^37–39^. In current study, we proposed two models for the GWAS of longitudinal trait which could fit the time-varied QTN effects and directly use the raw longitudinal records. This can fully avoid the necessity of transforming phenotypes into pseudo-phenotypes, such as EBVs^20^, DRPs^40^, or estimated residuals. The simulation results indicated that our proposed models could capture genetic differences varied in the entire process of the time period, thereby increasing the statistical power of QTN detection. Although pseudo-phenotypes were substitutions for longitudinal records, the scales of them would be changed^41^. Therefore, the QTN effects predicted by these pseudo-phenotypes methods were biased. This might not influence the significance test, as the scales of corresponding estimated errors would also change. However, the pseudo-phenotypes methods could not directly predict the true proportions of the phenotypic variance explained by QTNs. As our fGWAS-C and fGWAS-F models directly used raw phenotypes and achieved the most accurate estimate of the QTN effects, they could be used to predict QTN heritability in practice. Overall, the proposed random regression-based methods clearly outperformed other traditional methods validated by extensive simulations.

Among the traditional GWAS models, while no polygenetic effects were fitted to account for cryptic relationships between individuals, the GWAS-EBV-NP and GWAS-DRP-NP models resulted in high FPRs. DRPs had adjusted for parental average effect^23^. However, the cryptic relationships between individuals still existed when the EBVs were estimated from repeated measurements^22^. In the simulation study, our results indicated that GWAS-EBV-P and GWAS-DRP-P models had similar performance in controlling the FPRs. The GWAS-DRP-P models lowered the power in some degree in our simulation. This may be because the cryptic relationships were corrected twice. One is removing the parental average information from EBVs, the other is including the polygenetic effects into the GWAS model. The EBVs or DRPs had removed the environmental effects and combined the repeated phenotypic values into a single one for each individual, which resulted in a much smaller dimension of the mixed model equation, and thus was more computationally efficient and feasible. Therefore, the GWAS-EBV-P and GWAS-DRP-P models are still an appealing alternative for GWAS study of longitudinal data when the computational efficiency is the primary consideration.

Meanwhile, we applied our two models to a Chinese Holstein cattle data and the fGWAS-F model to the GAW18 data. The GWAS for milk production traits of Chinese Holstein population had been implemented by Jiang *et al*. with method similar to GWAS-EBV-P^20^. Here, we expanded the population size from 2,093 to 6,619. Furthermore, the Animal QTLdb had collected 4,585 QTLs (including the QTLs obtained from association analysis) for MY, FP and PP of dairy cattle since 1994, which made our study population be a suitable dataset for validating GWAS approaches for longitudinal traits. We mapped our significant SNPs to the Animal QTLdb and discovered that most of them (165/215) located within the reported QTL regions. Moreover, two novel regions (Chr5:93.49–94.65 MB for FP; Chr20: 44.16–45.87 MB for PP) contained several significant SNPs (15 SNPs for the former and 7 SNPs for the later) in relatively narrow segments, and they were potential candidate QTLs regions for milk production traits. The estimated curves of additive effects, dominance effects and QTN heritabilities of SNP ARS-BFGL-NGS-4939 (within the *DGAT1* region) for milk yield, fat percentage, and protein percentage were also predicted by our fGWAS-F model. The trend of these estimated curves implied that the genetic effects were not constant, and could depend on the data or environment.

For GAW18 dataset, Chen *et al*. did not find any significant SNPs using admixture mapping analysis^42^, and Chung and Zou found four significant SNPs with extended EMMA^1^ model^26^. These studies indicated that human blood pressure might have a complicated genetic background, and there might be no major genomic region affecting it. In our studies, totally eight significant SNPs were discovered by our fGWAS-F model, and their nearest genes participated in the biological processes of nucleotide-excision repair, blood coagulation, heart contraction and so on, which closely related to heart disease. These eight significant SNPs could be candidate associated loci for blood pressure.

Functional GWAS is not a novel conception, and has been proposed and carried out by Das *et al*.^43^. One of the key differences between their model and our proposed ones is that we divided the time-varied mean values for SNP genotypes into two parts, time-dependent population mean and SNP effects, instead of fitting them directly. In this way, our models can be easily implemented by the popular ASReml software. The more important difference is that time-varied polygenetic effects are fitted in our models to control the FPRs. We applied the fGWAS software by Das *et al*. to our simulated data. The resulted FPRs at tabulated thresholds of *p* value = 0.01 and 0.05 were 0.046 and 0.125, respectively. As indicated by Xu^44^ in GWAS of non-longitudinal traits, the model ignoring polygenic covariance structure merely performed well for the simple experiment with one QTN. However, the signals became very noisy for complex experiment with multiple QTNs. In practical situation, quantitative traits of interest are controlled by more than one QTN^45^. Therefore, it should be beneficial to include polygenic effect in the model.

The emerging next-generation sequencing technology impels us to find the “miss heritability” of complex traits. Along with the technological evolution, the availability of public data, such as the 1000 Genomes Project and 1000 Bull Genomes Project, provides opportunities to maximize the value of our existing data though genotype imputation. The number of variants can be increased and true QTNs may be located in this way. The genotype dosages (a continuous random variable between 0 and 2) can gain more powers than “best-guess” imputed genotype (genotypes with the highest probability) in GWAS^46^. Luckily, our fGWAS-C model can also be applied to genotype dosages. This cannot be achieved by the fGWAS software by Das *et al*.

As expected, our proposed fGWAS-C and fGWAS-F models showed obvious computational inefficiency as the dimension of the mixed model equation was larger than other models. When the relationship matrix is established based on pedigree, the computational burden is less challenging as the numerator relationship matrix is relatively sparse. The marker-based kinship matrix can reflect the relationship between individuals more precisely. For example, the relationship among full siblings will be the same based on pedigree, but can be distinguishable with genetic markers^47^. However, a dense marker-based kinship matrix will increase the computational burden heavily. Zhang *et al*. suggested that a compression approach, which was called compressed mixed linear model, would decrease the effective sample size by clustering individuals into groups^3^. Meanwhile, for population with unknown degree of genetic relationship, Kang *et al*. developed a procedure for estimating the contribution of the polygenetic effects to the phenotypes and the polygenetic effects were not needed to fit in the GWAS model if they were tested non-significant^2^. Both approaches can be incorporated to improve our proposed models in our future endeavors.

In conclusion, we proposed two models fGWAS-C and fGWAS-F using random regression for functional GWAS of longitudinal traits on a genome-wide scale. According to our simulation study results, the proposed models fitted longitudinal traits successfully and outperformed the models using EBVs, DRPs or estimated residuals as response variables. Using our proposed models, we have successfully found two novel regions which were significantly related with milking production traits for the Chinese Holstein data and some SNPs related with blood pressure for the GWA18 workshop dataset. Generally, functional GWAS models using random regression were useful and appealing in the GWAS for longitudinal traits.

## Materials and Methods

### General expression of the random regression model

The general expression of random regression model can be formulated as the time-dependent function:

1 $${y}_{i}(t)=\mu (t)+{a}_{i}(t)+{p}_{i}(t)+{e}_{i}(t),$$

where *y*_*i*_(*t*) is the phenotypic value of individual *i* at time *t*; *μ*(*t*) is the overall mean at time *t*; *a*_*i*_(*t*) and *p*_*i*_(*t*) are the time-varied additive genetic effect and permanent environmental effect respectively for individual *i*; *e*_*i*_(*t*) is the time-independent random residual for each measurement of individual *i* at time *t*. Here, *μ*(*t*), *a*_*i*_(*t*) and *p*_*i*_(*t*) can be denoted as the linear regression for a set of basis functions, *i.e*., splines or polynomials, below:

2 $$\mu (t)=\sum _{k={\rm{0}}}^{nf}{\beta }_{k}{\phi }_{k}(t),\,a{}_{i}(t)=\sum _{k={\rm{0}}}^{n{r}_{1}}{a}_{ik}{\phi }_{k}(t),\,p{}_{i}(t)=\sum _{k={\rm{0}}}^{n{r}_{2}}{p}_{ik}{\phi }_{k}(t),$$

where *nf*, *nr*_1_, and *nr*_2_ are the orders of corresponding basis functions; *φ*_*k*_(*t*) is the value of the *k*th basis function at time *t*; *β*_*k*_ is the *k*th fixed regression coefficient; *a*_*ik*_ and *p*_*ik*_ are the *k*th random regression coefficients for additive genetic effect and permanent environmental effect of the *i*th individual. The orders of different basis functions can be determined by model selection criteria (such as AIC and BIC) suggested by Das *et al*.^43^. The matrix form of (1) can be represented as:

3 $${\bf{y}}={\bf{X}}{\bf{b}}+{\bf{Q}}{\bf{a}}+{\bf{Z}}{\bf{p}}+{\bf{e}}.$$

Here, we assume there are *n* individuals and the number of records for individual *i* is *m*_*i*_ (*m*_*i*_ can be different for each individual), then the total number of records for all individuals is $m=\sum _{i=1}^{n}{m}_{i}$. Thus, **y** is a *m* × 1 vector of phenotypic values of all individuals; **b** is a [*n*(*nf* + 1)] × 1 vector of fixed regression coefficients; **a** is the vector of random regression coefficients for additive genetic effects with *nr*_1_ + 1 elements for each individual; **p** is the vector of random regression coefficients for permanent environmental effects with *nr*_2_ + 1 elements for each individual; **X**, **Q** and **Z** are the corresponding design matrices; **e** is the vector of random residuals.

For equation (3), we have the (co) variance matrices of all random effects:

$${\rm{var}}({\bf{a}})={\bf{A}}\otimes {\bf{G}},\,{\rm{var}}({\bf{p}})={\bf{I}}\otimes {\bf{P}},\,{\rm{and}}\,{\rm{var}}({\bf{e}})={\bf{I}}{\sigma }_{e}^{2}={\bf{R}}.$$

Here, **A** is the numerator relationship matrix based on pedigree information; **I** is the identity matrix; $\otimes$ is the Kronecker product; **G** is the variance–covariance matrix for random regression coefficients of additive polygenic effects with size of (*nr*_1_ + 1) × (*nr*_1_ + 1); **P** is the variance–covariance matrix of random regression coefficients for permanent environmental effects with size of (*nr*_2_ + 1) × (*nr*_2_ + 1); ${\sigma }_{e}^{2}$ is the residual variance. Therefore, the mixed model equations can be expressed as:

4 $$[\begin{array}{ccc}{{\bf{X}}}^{{\rm{T}}}{{\bf{R}}}^{-{\rm{1}}}{\bf{X}} & {{\bf{X}}}^{{\rm{T}}}{{\bf{R}}}^{-1}{\bf{Q}} & {{\bf{X}}}^{{\rm{T}}}{{\bf{R}}}^{-{\rm{1}}}{\bf{Z}}\\ {{\bf{Q}}}^{{\rm{T}}}{{\bf{R}}}^{-{\rm{1}}}{\bf{X}} & {{\bf{Q}}}^{{\rm{T}}}{{\bf{R}}}^{-{\rm{1}}}{\bf{Q}}+{{\bf{A}}}^{-{\rm{1}}}\otimes {{\bf{G}}}^{-{\rm{1}}} & {{\bf{Q}}}^{{\rm{T}}}{{\bf{R}}}^{-{\rm{1}}}{\bf{Z}}\\ {{\bf{Z}}}^{{\rm{T}}}{{\bf{R}}}^{-{\rm{1}}}{\bf{X}} & {{\bf{Z}}}^{{\rm{T}}}{{\bf{R}}}^{-{\rm{1}}}{\bf{Q}} & {{\bf{Z}}}^{{\rm{T}}}{{\bf{R}}}^{-{\rm{1}}}{\bf{Z}}+{\bf{I}}\otimes {{\bf{P}}}^{-1}\end{array}]\,[\begin{array}{c}\hat{{\bf{b}}}\\ \hat{{\bf{a}}}\\ \hat{{\bf{p}}}\end{array}]=[\begin{array}{c}{{\bf{X}}}^{{\rm{T}}}{{\bf{R}}}^{-{\rm{1}}}{\bf{y}}\\ {{\bf{Q}}}^{{\rm{T}}}{{\bf{R}}}^{-{\rm{1}}}{\bf{y}}\\ {{\bf{Z}}}^{{\rm{T}}}{{\bf{R}}}^{-{\rm{1}}}{\bf{y}}\end{array}].$$

### Statistical models for the GWAS of longitudinal data

Under the framework of the random regression model, we proposed two detection methods for the association analysis of longitudinal traits, *i.e*., functional GWAS model treating each SNP as the covariate (fGWAS-C), and functional GWAS model treating each SNP as the factor (fGWAS-F). To exploit the property of these two novel methods, several alternative models/strategies which used the EBVs, DRPs, or estimated residuals as the response variables for GWAS of longitudinal traits were also applied for extensive comparisons. Details of each model were specified below as well as listed in Table 2.

**Table 2:** The characters of fGWAS-C, fGWAS-F, GWAS-EBV-P, GWAS-EBV-NP, GWAS-DRP-P, GWAS-DRP-NP, and GWAS-Residual models.

Models	Response variable	SNP effect time dependence	SNP effect modeling	polygenetic effects (fit or not)
fGWAS-C	longitudinal records	time-dependent	covariate	YES
fGWAS-F	longitudinal records	time-dependent	factor	YES
GWAS-EBV-P	EBVs	time-independent	covariate	YES
GWAS-EBV-NP	EBVs	time-independent	covariate	NO
GWAS-DRP-P	DRPs	time-independent	covariate	YES
GWAS-DRP-NP	DRPs	time-independent	covariate	NO
GWAS-Residual	estimated residuals	time-independent	covariate	NO

#### fGWAS-C and fGWAS-F models

In our proposed fGWAS-C model, an additional fixed regression term is incorporated into equation (1) to explain effect of the SNP investigated:

5 $${y}_{i}(t)=\mu (t)+{x}_{i}SNP(t)+{a}_{i}(t)+p{e}_{i}(t)+{e}_{i}(t).$$

Here, *x*_*i*_ is a genotype indicator which is assigned 0, 1 and 2 for genotype *aa*, *Aa* and *AA*, respectively; *SNP*(*t*) represents the time-varied additive effect for each marker and can be expressed as linear regression for a set of basis functions as mentioned before:

6 $$SNP(t)=\sum _{k=0}^{nf}{\eta }_{k}{\varphi }_{k}(t),$$

where, ${\varphi }_{k}(t)$ is the value of the *k*th basis function at time *t*; ${\eta }_{k}$ is the *k*th fixed regression coefficient for additive SNP effect; *nf* is the order of basis functions for the time-varied SNP effect. For convenience, we define the same order of time-varied population mean and SNP effect in this model.

Similarly, the fGWAS-F model is formulated as:

7 $${y}_{il}(t)=\mu (t)+SN{P}_{l}(t)+{a}_{i}(t)+p{e}_{i}(t)+{e}_{il}(t),$$

where

8 $$SN{P}_{l}(t)=\sum _{k=0}^{nf}{\lambda }_{lk}{\varphi }_{k}(t){\rm{.}}$$

Here, *SNP*_*l*_(*t*) means time-varied effect for genotype *l* (*AA*, *Aa* and *aa*) of each marker and *λ*_*lk*_ is the *k*th fixed regression coefficient for genotype *l*. For fGWAS-F model, time-varied additive genetic effect, dominance genetic effect, and additive genetic variance of each SNP can be deduced as^45^:

9 $$\begin{array}{c}add(t)=\frac{SN{P}_{AA}(t)-SN{P}_{aa}(t)}{2},\,dom(t)=SN{P}_{Aa}(t)-add(t),\\ {\rm{and}}\,{\sigma }_{a,SNP}^{2}(t)=2pq{(add(t)+dom(t)(q-p))}^{2},\end{array}$$

where *p* and *q* are the allele frequencies for each locus.

#### GWAS-EBV-P and GWAS-EBV-NP models

Under such models, combined EBVs are firstly deduced through solving Equation (4). Therefore, the estimated additive genetic curve for individual *i* can be formulated as $\hat{a}{}_{i}(t)=\sum _{k=0}^{n{r}_{1}}{\hat{a}}_{ik}{\phi }_{k}(t)$. If we write:

10 $${\hat{{\bf{a}}}}_{i}={(\hat{a}_{i0}\,{}_{i1}\hat{a}\cdots {\hat{a}}_{in{r}_{1}})}^{{\rm{T}}},\,{{\bf{Q}}}_{t}^{{\rm{T}}}=({\phi }_{0}(t)\,{\phi }_{1}(t)\,\mathrm{...}\,{\phi }_{n{r}_{1}}(t)),\,{\rm{and}}\,{{\bf{Q}}}_{c}^{{\rm{T}}}=\sum _{t={t}_{\min }}^{{t}_{\max }}{{\bf{Q}}}_{t}^{{\rm{T}}},$$

where *t*_min_ and *t*_max_ are the first and last recording time of all individuals, then the accumulated EBV for each individual from *t*_min_ to *t*_max_ can be obtained as:

11 $$EB{V}_{i}=\sum _{t={t}_{\min }}^{{t}_{\max }}{\hat{a}}_{i}(t)=\sum _{t={t}_{\min }}^{{t}_{\max }}{{\bf{Q}}}_{t}^{{\rm{T}}}{\hat{{\bf{a}}}}_{i}={{\bf{Q}}}_{c}^{{\rm{T}}}{\hat{{\bf{a}}}}_{i.}$$

The accumulated EBVs are then used as the latent response variable in the GWAS-EBV-P model.

12 $${\bf{y}}={\boldsymbol{\mu }}+{\bf{W}}a+{\bf{Zu}}+{\bf{e}},$$

and GWAS-EBV-NP model

13 $${\bf{y}}={\boldsymbol{\mu }}+{\bf{W}}a+{\bf{e}}.$$

Here, **y** is defined as the n × 1 vector of EBVs for all individuals; **μ** is the population mean; **u** is the vector of polygenetic effects with multivariate normal distribution *MVN*(**0**, **A** ${\sigma }_{a}^{2}$), where **A** is the numerator relationship matrix and ${\sigma }_{a}^{2}$ is the additive genetic variance; **e** is the vector of random residuals with a multivariate normal distribution *MVN*(**0**, **I** ${\sigma }_{e}^{2}$); **Z** is the incidence matrix for polygenetic effects; *a* is the regression coefficient of EBVs on SNP genotypes and **W** is the vector of SNP genotypes coded as 0, 1, and 2.

#### GWAS-DRP-P and GWAS-DRP-NP models

We used DRPs instead of deduced EBVs as potential response variable in Equations 12 and 13, and the respective models were called GWAS-DRP-P and GWAS-DRP-NP. DRPs were derived from EBVs using the method proposed by Garrick *et al*. which allowed for the removal of the parental average information from EBVs^23^.

#### GWAS-Residual model

The GWAS-Residual model uses the adjusted estimated residuals as the response variable in the GWAS analysis. This model is similar to the genomewide rapid association using mixed model and regression (GRAMMAR) model^33^, which obtains residuals of all individuals adjusted for polygenetic effects and subsequently analyzes the association between these residuals and each SNP covariate using rapid least-squares methods.

In our study, the estimated residuals for multiple records corresponding to each individual were obtained by solving equation (4). Then, we averaged the estimated residuals of multiple records for each individual as the adjusted estimated residual, which was employed as the response variable for association analysis with the model similar to GWAS-EBV-NP or GWAS-DRP-NP.

### Hypothesis tests

For each SNP, the incremental Wald statistic implemented by ASReml^48^ was used to examine whether the SNP is associated with the trait. The Wald chi-squared statistic with a degree of freedom of *df*_w_ is given by:

$$W=\frac{R({\rm{full}}\,{\rm{model}})-R({\rm{reduced}}\,{\rm{model}})}{{\hat{\sigma }}_{e}^{2}}.$$

Here, $[R({\rm{full}}\,{\rm{model}})-R({\rm{reduced}}\,{\rm{model}})]$ denotes the difference between the reduction in the sums of squares^49^ (RSS) or models with and without SNP effect. The symbol *df*_*w*_ is degree of freedom for the SNP effect. For fGWAS-C model, *df*_*w*_ = *nf* + 1, and for fGWAS-F model, *df*_*w*_ = 2(*nf* + 1), where *nf* is the order of basis functions for the time-varied SNP effect as defined above. For other models defined above, *df*_*w*_ = 1. The symbol ${\hat{\sigma }}_{e}^{2}$ is residual variance estimated via residual maximum likelihood (REML) method.

### Simulations

We performed extensive simulations to systematically compare the performance of two random regression-based GWAS models (fGWAS-C and fGWAS-F) proposed here and other multiple-step traditional linear mixed models (GWAS-EBV-P, GWAS-EBV-NP, GWAS-DRP-P, GWAS-DRP-NP and GWAS-Residual) aforementioned. We evaluated statistical power, type-I error rate as well as the accuracy of SNP effect estimated for each GWAS method through 1,000 replication.

Population and genomic data were simulated with QMSim software^50^. The simulation started with a base population of 50 males and 50 females in generation −1,000, followed by 1,000 discrete historical generations (generation −1,000 to −1) with the same population size and an equal sex ratio. After 1,000 historical generations, the recent population was generated from generation −1 to generation 0 with population size expanded from 100 to 1,000 (500 males and 500 females). In the follow-up four recent generations (generation 1 to 4), 50 males were randomly selected from the last generation each mating with 500 females. Each female produced two offspring (one male and one female) at each recent generation. Finally, a total of 2,000 females from the last four recent generations were collected as the experimental population investigated. We determined 1,002 SNPs as the simulated genomic data, two of which were selected as independent target mutations. One contributed to the genetic variance (treated as the QTN) and the other had null effect on the longitudinal phenotype. These two SNPs were adopted to evaluate statistical powers and FPRs respectively across different models. The remaining 1000 SNPs were assigned the polygenic effects representing the genetic background of each individual, all genotypes of which were then removed in the final simulated data.

The longitudinal phenotype observations were simulated using self-developed C program. The detailed description was given in Supplementary Methods. In the simulation, heritability of simulated trait *h* ^2^ was set to 0.3 and heritability of functional QTN ${h}_{{\rm{QTN}}}^{2}$ (the proportion of phenotypic variance explained by the QTN) was set to different levels of 0.1%, 0.5%, 1% and 2%. The variances explained by the polygenetic and permanent environmental effect were scaled to achieve the preset heritability of the simulated trait.

In the simulation, the power and type-I error rate for each scenario were determined as the proportion of significant detections for functional QTN and null-effect SNP respectively among 1,000 replicates for each scenario.

### Real data analysis

Two real datasets, a Chinese Holstein cattle data and the Genetic Analysis Workshop 18 (GAW18) data, were used to further validate performance of our proposed models. The detailed illustrations of the real data was provided in Supplementary Methods. For simplicity and conciseness, we merely employed the fGWAS-F model in the real data of longitudinal traits. Legendre polynomials^51^ were used as the basis functions for the overall mean, additive genetic effect and permanent environmental effect. The orders of basis functions were evaluated based on the smallest AIC as well as BIC. As the effects contributed by most of SNPs were very small, we adopted the same variance components estimated by the reduced model of equation (1), which was then applied to the full GWAS model for testing each marker. This was similar to the strategy of Kang *et al*.^2^ and Zhang *et al*.^3^. The Bonferroni correction was used to control false-positive rates for Chinese Holstein cattle data. Therefore, the threshold for genome-wide significance was 0.05/N, where N was the number of SNPs to be tested. For the GAW18 data, we estimated *q* values for false discovery rates^52^ and a false discovery rate with *q* value of 0.05 was used as the threshold of the significant associations.

## Electronic supplementary material

Supporting Info

Supporting Info

Supporting Info

Supporting Info
