# Supplementary material for: Performance Gains in Genome-Wide Association Studies for Longitudinal Traits via Modeling Time-varied effects
Source: Sci Rep. 2017 Apr 4;7:590. doi: 10.1038/s41598-017-00638-2 (PMC5428860; doi:10.1038/s41598-017-00638-2)
Supplement: Supplementary file 1 — Supporting Info [file 41598_2017_638_MOESM1_ESM.docx]

**Performance Gains in Genome-Wide Association Studies for Longitudinal Traits via Modeling Time-varied effects**

Chao Ning^1#^, Huimin Kang^1#^, Lei Zhou^1^, Dan Wang^1^, Haifei Wang^1^, Aiguo Wang^1^, Jinluan Fu^1^, Shengli Zhang^1^ and Jianfeng Liu^1*^

^1^National Engineering Laboratory for Animal Breeding; Key Laboratory of Animal Genetics, Breeding and Reproduction, Ministry of Agriculture; College of Animal Science and Technology, China Agricultural University, Beijing, 100193, China.

*Corresponding author:

E-mail: [liujf@cau.edu.cn](mailto:liujf@cau.edu.cn).

^#^These authors contributed equally to this work.

**Supplementary Methods**

**Detailed description for phenotype simulation**

The recorded phenotypes of all individuals were simulated based on the model as follow:

$y_{i}\text{(}t\text{)}=\mu\text{(}t\text{)}+x_{i}SNP_{1}\text{(}t\text{)}+\sum_{j=2}^{1001} x_{i}SNP_{j}\text{(}t\text{)}+pe_{i}\text{(}t\text{)}+e_{i}(t)$ (S1)

Here, $x_{i}$ was a genotype indicator which is assigned 0, 1 and 2 for genotype *aa*, *Aa* and *AA*, respectively, and Wilmink polynomials^1^ were used as basis functions. The Wilmink polynomials were formulated as

$w\text{(}t\text{)}=a_{0}+a_{1}t+a_{2}e^{-0.05t}$.

The simulated trait was measured at several time points (*t*), with a range of [5, 305]. The first time point of measurement was sampled from a discrete uniform distribution$DU \left(5, 35\right)$, and subsequent time points were generated by adding intervals sampled from$DU \left(15, 36\right)$. The last time point was set to be not greater than 305. Let $\mathbf{w}_{t}$ be the vector of values for Wilmink polynomials’ basis functions (i.e., 1, *t*, and *e*^-0.05^*^t^*) at time *t*, then the cumulative vector from 5 to 305 was

$$\mathbf{w}^{\text{T}}=\sum_{t=5}^{305} \mathbf{w}_{t}^{\text{T}}=\sum_{t=5}^{305} \left[\begin{matrix} 1 & t & e^{-0.05t} \end{matrix}\right]=\left[\begin{matrix} 301 & 46655 & 15.97 \end{matrix}\right]$$

For time varied population mean, the vector of Wilmink polynomials’ coefficients were set to be $\left[\begin{matrix} 33.22 & -0.03647 & -20.96 \end{matrix}\right]$. For the SNP treated as the QTN, let the vector of three coefficients of Wilmink polynomials for additive effect curve be:

$a=\left[\begin{matrix} a_{0} & a_{1} & a_{2} \end{matrix}\right]^{\text{T}}$.

According to Falconer and Mackay ^2^, the variance–covariance matrix for the Wilmink polynomials’ coefficients of this SNP could be deduced as:

$\Sigma_{SNP_{1}}=\left[\begin{matrix} 2pqa_{0}^{2} & 2pqa_{0}a_{1} & 2pqa_{0}a_{2} \\ 2pqa_{0}a_{1} & 2pqa_{1}^{2} & 2pqa_{1}a_{2} \\ 2pqa_{0}a_{2} & 2pqa_{1}a_{2} & 2pqa_{2}^{2} \end{matrix}\right]=2pq\left[\begin{matrix} a_{0}^{2} & a_{0}a_{1} & a_{0}a_{2} \\ a_{0}a_{1} & a_{1}^{2} & a_{1}a_{2} \\ a_{0}a_{2} & a_{1}a_{2} & a_{2}^{2} \end{matrix}\right]$. (S2)

Where *p* and *q* were the allele frequencies for *A* and *a* alleles, respectively. The cumulative additive effect from time points 5 to 305 and corresponding explained variance for this SNP could be expressed as:

$a_{SNP_{1}}=\sum_{t=5}^{305} SNP_{1}\text{(}t\text{)}=\sum_{t=5}^{305} w_{t}^{\text{T}}a=w^{\text{T}}a$ (S3)

$\sigma_{SNP_{1}}^{2}=\text{var(}a_{SNP_{1}}\text{)}=\text{var(}w^{\text{T}}a\text{)}=w^{\text{T}}\text{var(}a\text{)}w=w^{\text{T}}\Sigma_{SNP_{1}}w$ (S4)

$\sigma_{SNP_{1}}^{2}=2pqa_{SNP_{1}}^{2}$ (S5)

Equation (S4) and (S5) can be proved to be equivalent. In the simulation, we fixed$\sigma_{SNP_{1}}^{2}$as 15,000. The SNP had allele frequencies of *p* = 0.42525 and *q* = 0.57475. Then, we had $a_{SNP_{1}}=175.21$. In order to achieve the above values of and $a_{SNP_{1}}$, the three coefficients of Wilmink polynomials for additive effect curve of this SNP was set as $\left[\begin{matrix} 0.04623 & 0.00327 & 0.5470 \end{matrix}\right]$.

The coefficients for additive effect curve of remaining 1,000 SNPs were randomly drawn from the multivariate normal distribution with

$MVN\left(\mathbf{0}, \left[\begin{matrix} 13.11 & \text{-0.2935E-01} & \text{-36.94} \\ \text{-0.2935E-01} & \text{0.1433E-03} & \text{0.8010E-01} \\ \text{-36.94} & \text{0.8010E-01} & 147.9 \end{matrix}\right]\right)$,

and the variance–covariance matrix and cumulative variance of each SNP were calculated using (2) and (4). Similarly, we obtained the coefficients for *p_i_*(*t*) by drawing from the multivariate normal distribution *MVN*(**0**, **Σ***_p_*). The residual variance for each measurement was $\sigma_{e}^{2}=16$. The cumulative phenotypic variance (*i.e.*, the variance for the accumulated phenotypic values from time points 5 to 305) was formulated as:

$\sigma_{y}^{2}=\sum_{j=1}^{1001} \sigma_{SNP_{j}}^{2}+\sigma_{pe}^{2}+301\sigma_{e}^{2}=\sum_{j=1}^{1001} W^{\text{T}}\Sigma_{SNP_{j}}W+W^{\text{T}}\Sigma_{pe}W+301\sigma_{\text{e}}^{2}$.

Then, the proportion of cumulative phenotypic variance explained by all the 1001 SNPs (i.e., heritability) and the SNP treated as QTN (SNP_1_) was:

$h^{2}=\frac{\sum_{j=1}^{1001} \sigma_{SNP_{j}}^{2}}{\sigma_{y}^{2}}\text{,} h_{QTN}^{2}=\frac{\sigma_{SNP_{1}}^{2}}{\sigma_{y}^{2}}$.

In the simulation, the *h*^2^ was 0.3 and$h_{QTN}^{2}$(heritability of the first SNP treated as QTN) was set to be 0.1%, 0.5%, 1% or 2%. For scenario of $h_{SNP_{1}}^{2}=0.1\%$, **Σ***_p_* had the following value:

$\sum_{pe}=\left[\begin{matrix} 194.07 & \text{-0.505} & \text{-18.702} \\ \text{-0.505} & 0.0033 & 0.0464 \\ \text{-18.702} & 0.0464 & 2.00 \end{matrix}\right]$.

The cumulative additive variance for the SNP treated as QTN and residual variance were constant, and the variance explained by the remaining 1000 SNPs and permanent environmental effect were scaled to achieve the different QTN heritabilities. One thousand replicates were generated and analyzed for each scenario.

**Chinese Holstein cattle data**

The original study population contained 9,615 Chinese Holsteins cattle genotyped with Illumina BovineSNP50v1 BeadChip (54,001 SNPs), Illumina BovineSNP50v2 BeadChip (54,609 SNPs) and GeneSeek Genomic Profiler HD (76,879 SNPs). The numbers of genotyped animals for the three versions of chips were 2,026, 4,996 and 2,593, respectively. All the SNPs located on sex chromosomes were removed due to the slightly lower imputation accuracies^3^. The individuals genotyped with GeneSeek Genomic Profiler HD were used as reference, and all others were imputed to this density using FImputev2.2^4^. Three quantitative traits including milk yield (MY), fat percentage (FP) and protein percentage (PP) of the first parity were analyzed in this study. The cows with less than six records and all the bulls were filtered out, which resulted in 6,619 cows with a total of 57,575 records. We removed the SNPs with minor allele frequency (MAF) less than 0.03 and those not passing the Hardy-Weinberg Equilibrium (HWE) test (*p*-value < 10^-6^), resulting in 71,624 SNPs for the subsequent GWAS analyses. For all of the GWAS models for Chinese Holstein data, an additional herd–test–date (HTD) fixed effect independent of time was included, and *μ*(*t*) was nested within each group of cows in the same region and of the similar age to fit the mean curve of this group. After model selection with AIC and BIC values, the model with a fifth-order Legendre polynomial for population mean, a third-order for additive genetic effects and a fifth-order for permanent environmental effects was best fit to the data for all the three traits. The fGWAS-F model with these combination of polynomials for different effects was used for the GWAS of the Chinese Holstein cattle data. The Bonferroni correction was used to control false-positive rates. Therefore, the threshold for genome-wide significance was 0.05/N, where N was the number of SNPs to be tested.

**GAW18 data**

GAW18 provides whole-genome sequence (WGS) data from a human pedigree-based sample to evaluate the developed GWAS models. The dataset included longitudinal measurements of systolic blood pressure (SBP) and diastolic blood pressure (DBP) of 835 individuals, and genotypes with 8,348,674 SNPs from the odd numbered autosomes (based on WGS data) for all individuals. In addition, age, sex, medication use and smoking status were also recorded in the dataset. The participants were examined for 1-4 times of SBP and DBP, and the majority of them had three measurements. Quality control of the genotype data was implemented similar to the procedure of Chinese Holstein cattle data. In order to reduce the computational burden, a pruned subset of 881,363 SNPs that were in approximately similar linkage equilibrium with each other were picked with PLINK^5^. We applied the fGWAS-F model to the two log-transformed phenotypes, log(SBP) and log(DBP). As higher order Legendre polynomial did not converge, the model with a second-order Legendre polynomial for all the time-varied effects was used to fit the data. For analysis model, medication use and smoking status were included as fixed effect independent of time and *μ*(*t*) was nested within sex to fit the mean curve of different groups of sex. We estimated *q* values for false discovery rates^6^ and a false discovery rate with *q* value of 0.05 was used as the threshold of the significant associations.

**Supplementary Table S1. Means, standard deviations (SD), and root-mean-square errors (RMSE) of estimated cumulative dominant genetic effect of the QTN by fGWAS-F model with various QTN heritabilities in the simulation study.**

| ***h*^2^_QTN_** | **Mean** | **SD** | **RMSE** |
| --- | --- | --- | --- |
| 0.1% | -1.00 | 160.41 | 160.33 |
| 0.5% | -0.71 | 75.05 | 75.01 |
| 1.0% | -4.34 | 54.82 | 54.96 |
| 2.0% | 0.56 | 41.58 | 41.57 |

**Supplementary Table S2. Akaike Information Criterion (AIC) and Bayesian Information Criterion (BIC) values for different orders of basis functions by reduced fGWAS-F model in the Chinese Holstein study.**

| order | | | MY^a^ | | FP^b^ | | PP^c^ | |
| --- | --- | --- | --- | --- | --- | --- | --- | --- |
| PM^d^ | AGE^e^ | PEE^f^ | AICs | BICs | AICs | BICs | AICs | BICs |
| 3 | 3 | 3 | 235996.3 | 236184 | 1836.86 | 2122.95 | -116961 | -116675 |
| 3 | 3 | 4 | 235049 | 235281.4 | 1883 | 2115.44 | -115776 | -115543 |
| 3 | 3 | 5 | 234581.3 | 234867.4 | 2288.07 | 2475.81 | -114271 | -114084 |
| 3 | 4 | 3 | 235573 | 235805.4 | 1897.51 | 2174.66 | -116957 | -116626 |
| 3 | 4 | 4 | 235240.7 | 235517.9 | 2068 | 2300.45 | -116031 | -115754 |
| 3 | 4 | 5 | 234601.9 | 234932.6 | 2099.52 | 2430.31 | -115365 | -115133 |
| 3 | 5 | 3 | 235322.9 | 235609 | 1949.11 | 2235.19 | -116530 | -116146 |
| 3 | 5 | 4 | 235019 | 235349.7 | 2071.35 | 2402.14 | -116389 | -116059 |
| 3 | 5 | 5 | 234894.6 | 235279 | 2580.47 | 2964.9 | -116025 | -115739 |
| 4 | 3 | 3 | 235081 | 235268.7 | 1898.77 | 2131.21 | -116929 | -116643 |
| 4 | 3 | 4 | 234935.9 | 235168.4 | 1982.37 | 2170.11 | -115828 | -115596 |
| 4 | 3 | 5 | 234508.1 | 234794.1 | 2134.91 | 2420.99 | -115590 | -115402 |
| 4 | 4 | 3 | 234903.3 | 235135.8 | 1849.87 | 2082.3 | -116664 | -116333 |
| 4 | 4 | 4 | 234944.8 | 235221.9 | 1862.44 | 2139.57 | -116108 | -115876 |
| 4 | 4 | 5 | 234547.5 | 234878.3 | 2134.28 | 2465.06 | -116049 | -115772 |
| 4 | 5 | 3 | 234758.1 | 235044.2 | 1770.16 | 2100.94 | -116662 | -116277 |
| 4 | 5 | 4 | 234882.5 | 235213.3 | 1776.62 | 2062.7 | -116493 | -116207 |
| 4 | 5 | 5 | 234652.3 | 235036.7 | 2273.99 | 2658.4 | -116482 | -116151 |
| 5 | 3 | 3 | 234608.7 | 234796.4 | 1800.83 | 2033.25 | -116939 | -116653 |
| 5 | 3 | 4 | 234471.8 | 234704.2 | 1868.12 | 2055.85 | -116786 | -116553 |
| 5 | 3 | 5 | 234410.1 | 234696.2 | 2202.86 | 2488.92 | -116467 | -116279 |
| 5 | 4 | 3 | 234443.6 | 234676.1 | 1745.64 | 2022.76 | -116805 | -116572 |
| 5 | 4 | 4 | 234471.6 | 234748.7 | 1756.43 | 1988.85 | -116818 | -116541 |
| 5 | 4 | 5 | 234431.5 | 234762.3 | 2201.76 | 2532.52 | -116648 | -116317 |
| 5 | 5 | 3 | 234371.6 | 234657.7 | 1710.18 | 1996.25 | -116972 | -116686 |
| 5 | 5 | 4 | 234404.8 | 234735.6 | 1713.95 | 2044.71 | -116963 | -116633 |
| 5 | 5 | 5 | 234466 | 234850.4 | 2242.56 | 2626.96 | -116580 | -116196 |

^a^MY: milk trait

^b^FP: fat percentage

^c^PP: protein percentage

^d^PM: population mean

^e^AGE: additive genetic effect

^f^PEE: permanent environmental effect

**
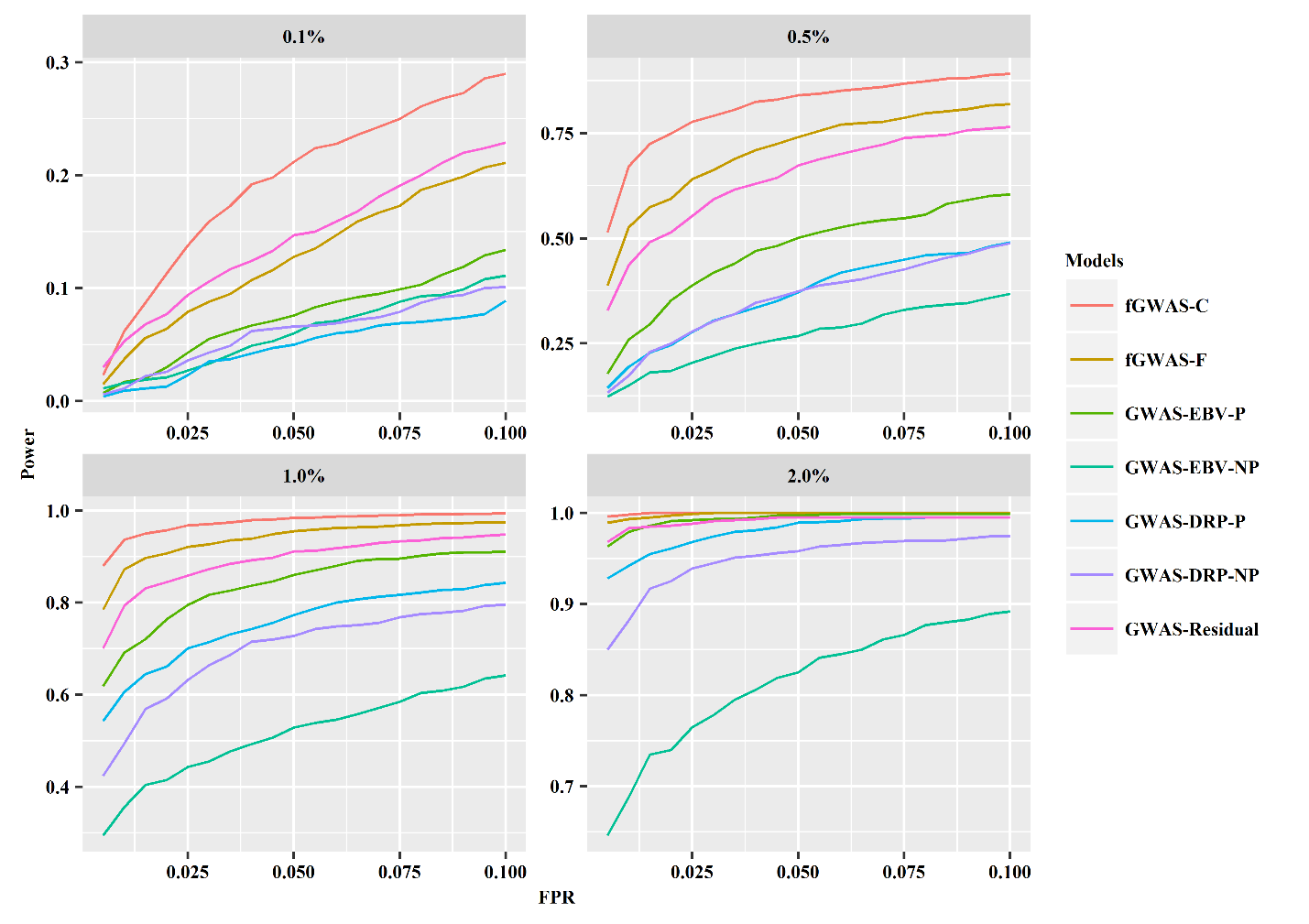
**

**Figure S1. Statistical powers of four alternative QTN heritabilities plotted against false positive rates (FPRs) for seven different GWAS models.**


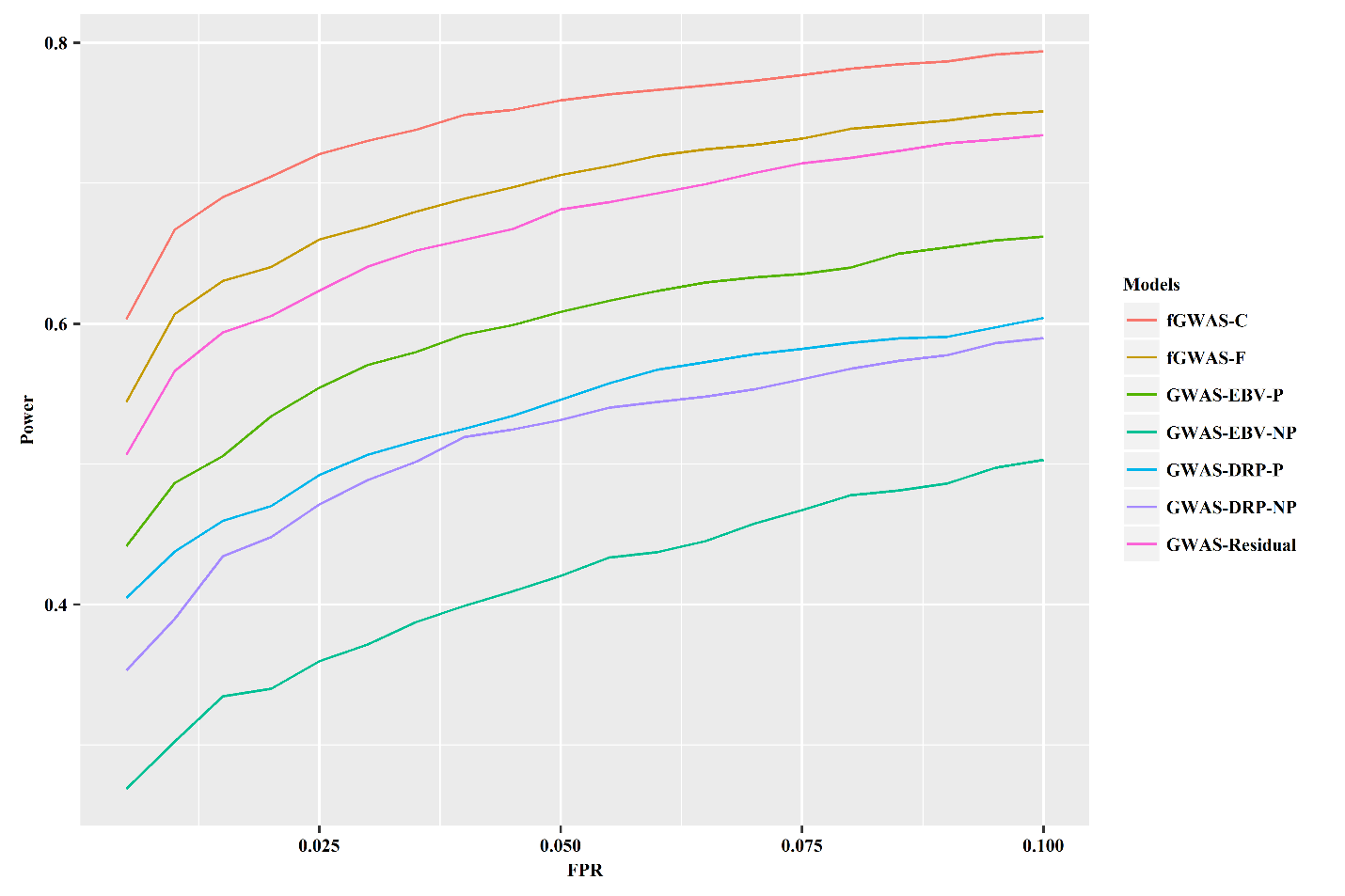


**Figure S2. The average statistical powers of four alternative QTN heritabilities plotted against false positive rates (FPRs) for seven different GWAS models.**


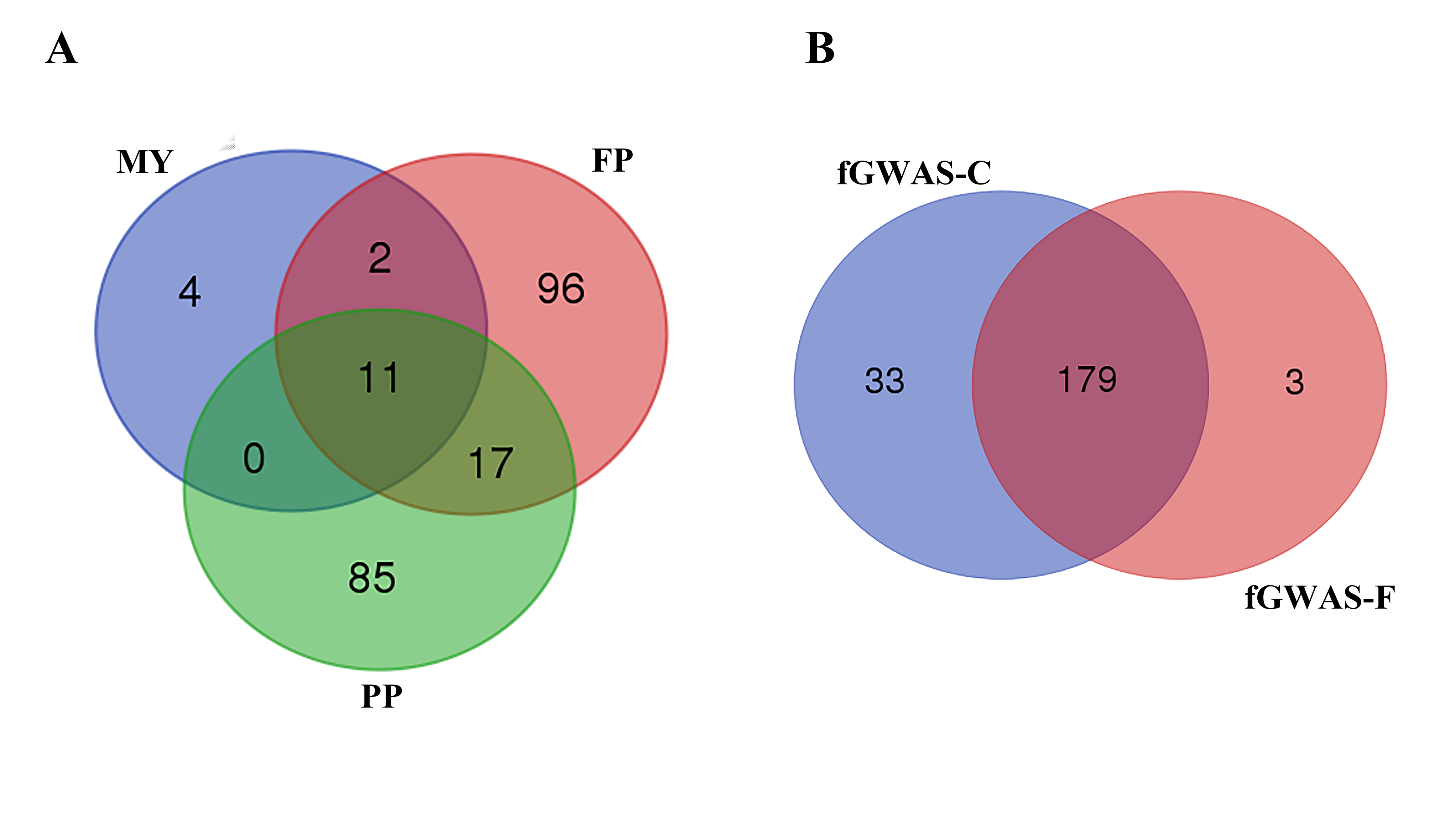


**Figure S3.** **Venn diagram showing the 215 significant SNPs for three traits of Chinese Holstein cattle data by the fGWAS-C and fGWAS-F models.**


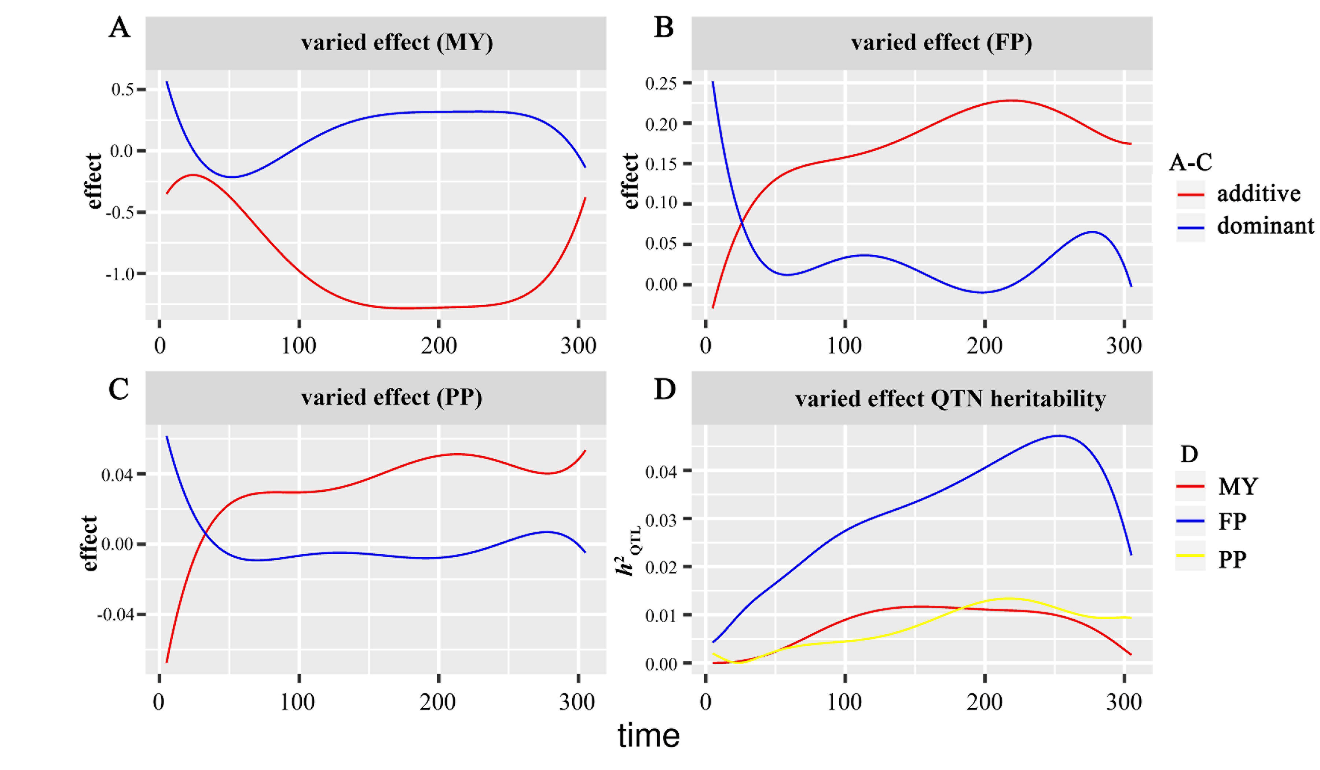


**Figure S4. The estimated curves of additive effects, dominance effects and QTN heritabilities of SNP ARS-BFGL-NGS-4939 (within the *DGAT1* region) for milk yield (MY), fat percentage (FP), and protein percentage (PP) by the fGWAS-F model for the Chinese Holstein cattle data.**

Figures 6-A to 6-C showed the curves of additive effects (red) and dominance effects (blue) over time-points for MY, FP, and PP, respectively. Figure 6-D showed the curves of QTN heritabilities (red for MY, blue for FP, and yellow for PP).

**Reference:**

1. Wilmink, J. Adjustment of test-day milk, fat and protein yield for age, season and stage of lactation. *Livest Prod Sci* **16,** 335-348 (1987).

2. Falconer, D. & Mackay, T. Introduction to Quantitative Genetics Ch. 18 (Longman, New York, 1981).

3. Hickey, J.M. & Kranis, A. Extending long-range phasing and haplotype library imputation methods to impute genotypes on sex chromosomes. *Genet Sel Evol* **45,** 1 (2013).

4. Sargolzaei, M., Chesnais, J.P. & Schenkel, F.S. A new approach for efficient genotype imputation using information from relatives. *BMC Genomics* **15,** 478 (2014).

5. Purcell, S. *et al.* PLINK: a tool set for whole-genome association and population-based linkage analyses. *Am J Hum Genet* **81,** 559-75 (2007).

6. Benjamini, Y. & Hochberg, Y. Controlling the false discovery rate: a practical and powerful approach to multiple testing. *J R Stat Soc B* **57,** 289-300 (1995).
